# Does gender inequality matter for access to and utilization of maternal healthcare services in Bangladesh?

**DOI:** 10.1371/journal.pone.0257388

**Published:** 2021-09-16

**Authors:** Firoz Ahmed, Fahmida Akter Oni, Sk. Sharafat Hossen

**Affiliations:** Economics Discipline, Social Science School, Khulna University, Khulna, Bangladesh; University of Southern Queensland, AUSTRALIA

## Abstract

There is a high prevalence of gender gap in Bangladesh which might affect women’s likelihood to receive maternal healthcare services. In this backdrop, we aim to investigate how gender inequality measured by intrahousehold bargaining power (or autonomy) of women and their attitudes towards intimate partner violence (IPV) affects accessing and utilizing maternal health care services. We used Bangladesh Demographic and Health Survey (BDHS) data of 2014 covering 5460 women who gave birth at least one child in the last three years preceding the survey. We performed logistic regression to estimate the effect of women’s autonomy and their attitude towards IPV on access to and utilization of maternal healthcare services. Besides, we employed different channels to understand the heterogeneous effect of gender inequality on access to maternal healthcare services. We observed that women having autonomy positively influenced attaining five required antenatal care (ANC) services (AOR: 1.17; 95% CI: 0.98–1.41) and women’s negative attitudes towards IPV were positively associated with five ANC services (AOR: 1.42; 95% CI: 1.02–1.97), sufficient ANC visits (COR: 1.55; CI: 1.19–2.01), skilled birth attendant (SBA) (AOR: 1.43; 95% CI: 1.05–1.94) and postnatal care (PNC) services (AOR: 1.44; 95% CI: 1.12–1.84). Besides, rural residency, religion, household wealth, education of both women and husband were found to have some of the important channels which were making stronger effect of gender inequality on access to maternal healthcare services. The findings of our study indicate a significant association between access to maternal healthcare services and women’s autonomy as well as attitude towards IPV in Bangladesh. We, therefore, recommend to protect women from violence at home and mprove their intrahousehold bargaining power to increase their access to and utilization of required maternal healthcare services.

## Background

Globally, access to and utilization of maternal healthcare services is considered as an important predictor to reduce maternal mortality [1]. Higher maternal deaths are the results of lack of access to proper healthcare and emergency services during and after pregnancy [2]. Moreover, the risks associated with pregnancy and delivery systems are the major causes of maternal mortality, which is severe, especially in developing countries [3]. However, access to and utilization of maternal healthcare services, including antenatal care service [4], assistance from skilled professionals during childbirth [5], access to emergency obstetric care [6], and appropriate postpartum care [7], can raise the probability of smooth and safe motherhood [8]. According to an estimation by World Health Organization (WHO) in 2013, globally, a total of 289,000 women died during their pregnancy and childbirth [9]. In Bangladesh, the maternal mortality rate is declining over time, but the rate is relatively higher compared to other South Asian countries [1]. According to Bangladesh Maternal Mortality and Health Care Survey (BMMS) 2010, the country experienced around 40% decline in maternal mortality between 2001 and 2010 [10]. Despite some positive changes, there is a limitation of providing universal access to maternal healthcare services in Bangladesh [11].

In recent times, the research agenda on maternal healthcare services focus on gender inequality at household level in achieving their right share of assets, capacity to raise their voice, and autonomy in decision making process [1, 2, 12]. Gender inequality (or equality) is often identified as a multidimensional and influential issue to women’s healthcare utilization in various ways [13]. The term ‘gender inequality’ is measured by different dimensions including economic, social, and political participation of women [14]. Even, there are multiple social and economic variables related to gender inequality. The indicators of gender inequality or gender gap play negative roles for the development of women’s capabilities, their freedom of choice, and self-esteem [13]. The autonomy of women in the household ensures their equal rights. Moreover, the improved bargaining power (autonomy) within household represents more gender equality in decision making and eventually, it shapes the attitude of women towards intimate partner violence (IPV) [15–17]. In the context of Bangladesh, women’s autonomy in intrahousehold decision making leads to a lower risk of IPV [18–20]. The linkage between women’s decision making power and attitude towards IPV can be explained by the fact that women with more autonomy in decision making pose negative attitudes towards IPV [17, 19]. Gender norms towards IPV represent self-esteem of women as well women’s personal choices towards their own life, and if women justified IPV then this can be defined as low self-esteem and low status of women in their households [21, 22]. Gender gap in decision making, roles and rights in the households, and self-esteem of women compared to their husbands represents gender inequality and disempowerment. Therefore, the combination of women’s autonomy or independence on intrahousehold decision making and their attitude toward IPV is used to measure gender equality or empowerment [15, 21–23].

Studies found that women’s autonomy was positively related to use of maternal healthcare services [24]. Besides, the changing gender norms also help in getting access to and use of maternal healthcare services when needed [5, 13, 19]. In many low- and middle-income countries, women, during pregnancy and childbirth, do not get access to maternal healthcare services due to the persistent gender inequality [25–27]. The differences in autonomy, domination of males over females, and gender-based violence are some of the deterrence revealed in the context of gender inequalities that limit the access to healthcare facilities of women [28–30]. IPV is often shaped by gender norms and communities’ ethics that places women in an inferior position compared to men [31].

**Fig 1:**
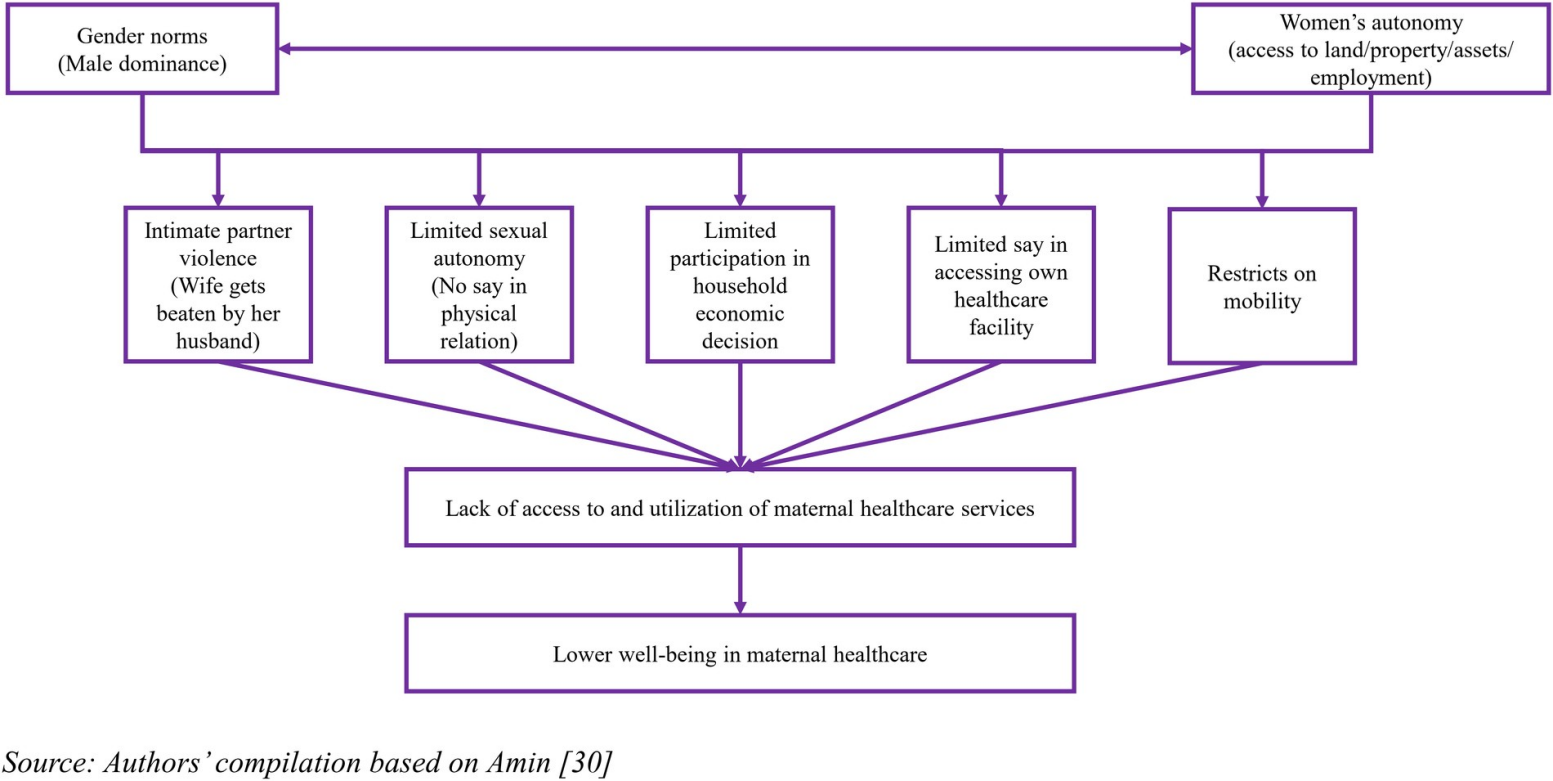
Pathways explaining how gender inequality shapes maternal healthcare services.

The detailed pathway of how gender-based inequality at the household level shapes the well-being of women to achieve quality in healthcare services is depicted in Fig 1. However, there has been little discussion on gender inequality and maternal well-being in quality healthcare in Bangladesh based on the dimensions mentioned earlier in the pathway. Therefore, we aim to investigate the linkage between gender inequality and access to and utilization of maternal healthcare services in Bangladesh.

## Materials and methods

### Data and sampling

We used Bangladesh Demographic and Health Survey (BDHS) data of 2014 covering 17,863 women of reproductive age (15–49 years) [32]. This is nationally representative and comprehensive data covering multiple issues related to maternal and child health status. The standard demographic and health survey (DHS) dataset can be obtained from the DHS program website, detailed path is mentioned in the data availability section. This is a good source of data with high quality and standard to conduct a study on social and health-related issues [33]. The details of the sampling and survey strategies are discussed in several papers [32, 34]. To address the study objectives, we used the children’s recode (KR) data file (file name—BDKR72FL). This dataset contains information on women (mothers) of each of these children born in the last five years preceding the survey and covers around 7,886 women. The information is mainly on maternal and newborn health which includes antenatal care, delivery, postnatal care, and women’s individual as well as household characteristics. For convenience, the study population comprised of women who gave birth at least one child in the last three years preceding the survey. Among the total of 7,886 women from this dataset, 5,460 women were found to be eligible to be included in this current study as the target respondents. To address the objective, we extracted information on socioeconomic characteristics of the respondents and their husbands, access to and utilization of maternal healthcare services, women’s autonomy and attitude towards IPV, and the household level data (e.g. wealth status of the household) from the same data file (children’s recode (KR) data—BDKR72FL).

### Outcome variables

Access to and utilization of antenatal care (ANC) service during pregnancy, skilled birth attendant (SBA) during delivery and access to postnatal care (PNC) were taken into consideration to measure maternal healthcare services. While estimating access to and utilization of ANC, we consider at least four ANC visits (or ANC visit ≥ 4) as sufficient and termed as ‘yes’ as opposed to insufficient ANC visits (ANC visit < 4) termed as ‘no.’ Though the recent 2016 WHO model recommends eight ANC visits, the country guideline of Bangladesh still promotes at least four ANCs to ensure sufficient ANC coverage [35, 36]. Therefore, we used at least four ANC visits (or ANC visits ≥ 4) to indicate the sufficient number of ANC visits. Along with the number of ANC visits, five different types of ANC services, including measurement of weight and blood pressure, assessment of urine and blood samples, and an ultrasonogram, were considered to define quality ANC services. We considered these five components of ANC services to understand whether sufficient ANC visits ensure proper monitoring of women to assess complications during pregnancy. By using these five required ANC components, we covered only access to and utilization of clinical examination and laboratory testing services and we excluded the information provision. If women reported yes in all five ANC services, then the response was coded as ‘yes’ that ensures quality ANC services while the inferior ANC service was considered as ‘no’ when the respondent did not get the five required ANC services. During the time of giving birth, women require SBA, such as a qualified doctor, trained nurse, or midwife. If the women give birth under the care of professional and trained birth attendants in–public or private hospital/community clinic or NGO static clinic–it was counted as ‘yes,’ when the delivery was carried out at home under unskilled or traditional birth attendant (TBA) the response was coded as ‘‘no’. PNC is categorized as ‘yes’ if the respondents received any type of postnatal services after delivery, and it was ‘no’ when the respondents did not receive any post-delivery checkup.

### Explanatory variables

In our study, we incorporated two sets of explanatory variables. The first set of explanatory variables includes women’s autonomy constructed from their intrahousehold decision making power in large purchases, own healthcare and own mobility (visit to family or relatives), and attitude towards intimate partner violence (IPV), such as justification of beating by their husbands while a woman goes out without having permission from her husband, neglects children at home, argues with husband, refuses to have sexual intercourse with her husband, burns food while cooking following some literature [16, 21–23]. Women’s autonomy is coded as ‘yes’ if at least two decisions of the three are taken by a woman alone or jointly with her husband as opposed to ‘no’ if the decisions taken in a similar way are less than two or a woman has no role in making household decisions. While the attitude towards IPV is coded as ‘yes’ if a woman disagrees more than one of five questions (*i*.*e*., *(i) goes out without telling her husband; (ii) neglects the children; (iii) argues with her husband; (iv) refuses to have sex with him; (v) burns the food*) related to her attitude towards IPV, and it was ‘no’ for otherwise. When a woman agrees that her husband can hit her for the above-mentioned reasons, this is termed as positive attitudes towards IPV as opposed to disagree for negative attitudes towards IPV. The second set of explanatory variables is related to respondents’ demographic and socioeconomic characteristics, including age, education, exposure to media (e.g., newspaper and/or television), place of residence, religion, wealth status of the household, last child’s birth order, and earlier experiences in pregnancy related complications.

### Analytical framework

In this study, we used descriptive statistics to reveal the socioeconomic and demographic characteristics of the respondents. In addition, we used logistic regression to estimate how women’s autonomy, attitude towards IPV, and other socioeconomic and demographic features influenced their access to and utilization of five ANC services, ANC visits, SBA, and PNC services. To estimate the factors of access to and utilization of maternal healthcare services, we relied on four outcome variables mentioned above. Both the simple and multivariate logistic regression models were constructed to examine the unadjusted and adjusted effects of women’s autonomy and their attitudes towards IPV on access to and utilization of maternal healthcare services. Therefore, we estimated two separate logistic regressions for each of the four outcome variables at 5% level of statistical significance. The study results were reported in both the unadjusted or crude odds ratio (COR) and adjusted odds ratio (AOR) with 95% confidence interval (CI). Before estimating four separate multivariate logistic regressions, we started to estimate five ANC services as the outcome variable and tried to find out the effects of different variables adding them stepwise and check their goodness of fit. The reason for stepwise adding explanatory variables is to check how they raised the goodness of fit in the estimation. Moreover, this procedure also helped to assess the unadjusted effect of each variable. However, we have not reported the results in our results section for the sake of brevity.

To better understand the effect of gender inequality on access to and utilization of five ANC services as a representation of maternal healthcare services, we focused on six different types of heterogeneous channels. These channels included their place of residence, religion, education of women, education of their husbands, watch TV, and wealth status of households. Considering these heterogeneous channels, we tried to find the role of gender inequality on access to five ANC services using both the simple and multivariate logistic regressions to estimate the unadjusted and adjusted effect for split sample. For example, we split the sample in rural and urban to address the heterogeneity by their place of residence. Finally, we also checked the effect of heterogeneous characteristics of women on their access to and utilization of maternal healthcare services using logistic regression. To examine the heterogeneous effect of the covariates on five ANC services, we relied on the interaction between women’s residence and education level, between women’s and husband’s education level, and between women’s residence and watch TV. For example, how far did access to information through watching TV affect maternal health care services when they differ by the location of residence (rural/urban)? Also, how did women’s education matter for their maternal healthcare services when their residence differs (rural/urban)? To check the heterogeneity through interaction effect, we used only five ANC services as the outcome variable as a representation of overall maternal healthcare services.

The BDHS sample was non-proportional in terms of urban-rural population distribution. Therefore, all frequency distributions were weighted while the survey command (svy) in STATA was used to adjust for the complex sampling structure of the data in the regression analyses to enable generalization of results to the eligible population, women who gave birth at least one child in the last three years preceding the survey. The data were analyzed using STATA version 13.0 for Windows.

### Ethical issues

We used BDHS 2014 data, which maintains strict ethical standards for protecting the privacy and confidentiality of the respondents during data collection. Moreover, procedures and questionnaires used for the BDHS 2014 were reviewed and approved by the ICF Institutional Review Board. Therefore, no further ethical approval is necessary since this study was based on publicly available data with no identifiable information.

## Results of the study

### Summary statistics of outcomes and explanatory variables

Our study considered a total of 5,460 women who experienced childbirth at least once in the last three years preceding the survey. Table 1 shows the distribution of the respondents across different socioeconomic and demographic features, and maternal healthcare services. We observed that about 42.25% of the respondents received the required ANC services, 31.18% attained sufficient ANC visits, 37.63% received SBA during delivery and 64.46% received the services of postnatal checkup (Panel A in Table 1). We also found that around 58.79% of the respondents had autonomy and 83.5% had negative attitudes towards IPV (Panel B in Table 1). Most of the respondents were Muslims (91.94%), nearly 75% lived in rural areas and 42.86% of them were poor (Panel C in Table 1). One of the interesting findings is that compared to their husbands the respondents were relatively more educated. For instance, 15.19% of the women were not educated which is lower than their husbands with no education (25.06%).

**Table 1:** Summary statistics of the outcome variables and explanatory variables.

Variables	Category	Frequency (N)	Percentage (%)
***Panel A*: *Outcome variables***			
Access to five ANC services	Yes	1491	42.25
	No	2038	57.75
Sufficient ANC visits (≥ 4)	Yes	1401	31.18
	No	3092	68.82
Receiving service from SBA	Yes	1777	37.63
	No	2947	62.38
Receiving PNC service	Yes	2897	64.46
	No	1597	35.54
***Panel B*: *Characteristics related to respondents***			
Having autonomy	Yes	3172	58.79
	No	2223	41.21
Attitude towards IPV	Negative	4559	83.50
	Positive	901	16.50
Age	≤ 20	1398	25.61
	21–30	3240	59.34
	≥ 31	822	15.05
Education	No education	829	15.19
	Primary	1550	28.39
	Secondary	2560	46.89
	Higher	520	9.53
Last child’s birth order	1	2269	41.56
	2–3	1601	29.32
	≥4	1590	29.12
Had pregnancy complications	Yes	1645	46.66
	No	1880	53.34
Reading newspaper	At least once in a week	285	5.23
	Not at all	5175	94.77
Watching television (TV)	At least once in a week	2569	47.06
	Not at all	2891	52.94
Education of husband	No education	1368	25.06
	Primary	1660	30.40
	Secondary	1683	30.82
	Higher	749	13.72
***Panel C*: *Household level characteristics***			
Religion	Muslim	5020	91.94
	Others	440	8.07
Place of residence	Rural	4088	74.87
	Urban	1372	25.13
Wealth index	Poor	2340	42.86
	Middle	2105	38.55
	Rich	1015	18.59

### Effect of gender inequality on access to and utilization of maternal healthcare services

We explored how women’s access to and utilization of maternal healthcare services differ depending on existing gender inequality within their household. To ensure effective service during pregnancy, women should have access to all five ANC services and sufficient ANC visits (visits ≥ 4) to ensure smooth and risk-free delivery. From the estimation, we observed that women with higher inequality were deprived of getting access to antenatal care services. Women’s autonomy and attitude towards IPV were associated with required ANC services and sufficient ANC visits. The unadjusted estimation revealed that women having autonomy in decision making were 1.20 (95% CI: 1.01–1.43) times more likely to receive five ANC services and women with negative attitudes towards IPV were 1.93 (95% CI: 1.46–2.56) times more likely to receive five ANC services (Model 1 in Table 2). Moreover, women with negative attitudes towards IPV were 1.55 (95% CI: 1.19–2.01) times more likely to attain sufficient ANC visits (Model 3 in Table 2). After adjusting for socioeconomic and demographic features, we found that women having autonomy in decision making were 1.17 (95% CI: 0.98–1.41) times more likely to receive five ANC services (Model 2 in Table 2). While women’s negative attitudes towards IPV (which represents equality) were positively associated with women’s access to five required ANC services. We found that women with negative attitudes towards IPV were 1.42 (95% CI: 1.02–1.97) times more likely to receive five ANC services. From our result, it is evident that women who enjoyed equality in terms of decision making and attitude towards IPV were more likely to receive required ANC services to avoid pregnancy-related complications.

**Table 2:** Factors influencing access to and utilization of five ANC services and ANC visits.

Variables	Five ANC Services						ANC visits (≥ 4 times)					
	Model 1			Model 2			Model 3			Model 4		
	COR	95% CI	*P value*	AOR	95% CI	*P value*	COR	95% CI	*P value*	AOR	95% CI	*P value*
Have autonomy [*Ref*. *No*]	1.20**	1.01–1.43	0.041	1.17*	0.98–1.41	0.087	1.13	0.96–1.32	0.151	1.06	0.89–1.27	0.504
Negative attitude towards IPV [*Ref*. *No*]	1.93***	1.46–2.56	0.000	1.42**	1.02–1.97	0.036	1.55***	1.19–2.01	0.001	0.98	0.73–1.33	0.921
Age [*Ref*. *age ≤ 20*]												
*21–30*				1.40**	1.06–1.85	0.018				0.94	0.71–1.24	0.667
*≥ 31*				2.00***	1.22–3.28	0.006				0.93	0.64–1.34	0.690
Education [*Ref*. *Illiterate*]												
*Primary education*				1.23	0.85–1.78	0.275				1.13	0.70–1.84	0.615
*Secondary education*				1.55*	0.93–2.58	0.091				1.51**	1.04–2.18	0.030
*Higher education*				1.80**	1.04–3.12	0.037				1.97***	1.21–3.21	0.007
Last child’s birth order [*Ref*. *one child*]												
*2–3*				0.81	0.60–1.09	0.161				1.04	0.81–1.34	0.736
*≥ 4*				0.68**	0.50–0.93	0.017				0.87	0.66–1.14	0.316
Had pregnancy complications [*Ref*. *No*]				1.91***	1.56–2.33	0.000				1.89***	1.54–2.33	0.000
Read newspaper at least once in a week [Ref. No]				1.44	0.90–2.33	0.130				1.53*	0.93–2.53	0.097
Watch TV at least once in a week [Ref. No]				1.24*	0.98–1.56	0.073				1.35***	1.09–1.67	0.007
Husband’s Education [*Ref*. *Illiterate*]												
*Primary education*				1.29	0.81–2.04	0.286				1.11	0.83–1.49	0.479
*Secondary education*				1.58***	1.14–2.21	0.007				1.11	0.74–1.68	0.603
*Higher education*				2.42***	1.71–3.41	0.000				1.51**	1.05–2.17	0.026
Muslim [*Ref*. *others*]				0.81	0.51–1.28	0.359				0.90	0.65–1.27	0.559
Live in rural area [Ref. *Urban*]				0.92	0.71–1.19	0.532				0.78*	0.61–1.00	0.053
Wealth Status [*Ref*. *Poor*]												
*Middle*				1.85***	1.43–2.40	0.000				1.13	0.82–1.55	0.456
*Rich*				4.25***	2.88–6.29	0.000				1.57**	1.09–2.27	0.017
Constant	0.37***	0.27–0.51	0.000	0.09***	0.04–0.19	0.000	0.29***	0.23–0.37	0.000	0.28***	0.16–0.50	0.000
Observations	3,494			3,492			4,438			3,492		

Note

*** p<0.01

** p<0.05

* p<0.1.

In addition to gender inequality, education of women and their husband was positively associated with ANC services and sufficient ANC visits. Compared to women with no education, women having secondary and higher education were more likely to attain all five ANC services and more than four ANC visits. For example, compared to the women with no education, women having higher education were 1.80 (95% CI: 1.04–3.12) times more likely to get five ANC services (Model 2 in Table 2) and 1.97 (95% CI: 1.21–3.21) times more likely to attain sufficient ANC visits (Model 4 in Table 2). Moreover, husband’s education has a profound role in shaping access to five ANC services as well as sufficient ANC visits. Compared to women with no educated husband, women having higher educated husbands were 2.42 (95% CI: 1.71–3.41) times more likely to receive five ANC services and 1.51 (1.05–2.17) times more likely to receive sufficient ANC visits. Women living in rural areas were 22% (95% CI: 0.61–1.00) less likely to attain sufficient ANC visits.

Exposure to media also affected the access to five ANC services and sufficient ANC visits. Women who read newspapers were 1.53 (95% CI: 0.93–2.53) times more likely to receive sufficient ANC visits. Besides, women who watched television (TV) at least once a week were 1.24 (95% CI: 0.98–1.56) times more likely to receive the required ANC services and 1.35 (95% CI: 1.09–1.67) times more likely to attain sufficient ANC visits. Expectedly, we observed that compared to poor households, women from households with middle wealth were 1.85 (95% CI: 1.43–2.40) times more likely to get the required five ANC services, while women from rich households were 4.25 (95% CI: 2.88–6.29) times more likely to attain five ANC services and 1.57 (95% CI: 1.09–2.27) times more likely to attain sufficient ANC visits. Previous pregnancy complications among women were positively associated with required ANC service and sufficient ANC visits. Moreover, age of the women is positively linked with access to ANC services. One of the interesting results of our study is that the last child’s birth order significantly affected women’s access to five ANC services. Women with higher birth orders of their last child were less likely to receive the required five ANC services during their most recent pregnancy.

The results also indicate that gender inequality significantly affected access to SBA and PNC. The crude analysis revealed that women having negative attitudes towards IPV were 2.11 (95% CI: 1.67–2.65) times more likely to receive assistance from SBA (Model 1 in Table 3) and 1.83 (95% CI: 1.49–2.25) times more likely to receive PNC (Model 3 in Table 3). The results from adjusted analysis, we observed that women who had negative attitudes towards IPV were 1.43 (95% CI: 1.05–1.94) times more likely to receive assistance from SBA (Model 2 in Table 3) and 1.44 (95% CI: 1.12–1.84) times more likely to receive PNC (Model 4 in Table 3). Women living in rural areas were 33% (95% CI: 0.51–0.87) less likely to receive assistance from SBA and 36% (95% CI: 0.45–0.93) less likely to get PNC services. The respondents and their husband’s education were positively associated with SBA and PNC services. Compared to no educated women, women having secondary education were 1.81 (95% CI: 1.22–2.70) times more likely to receive assistance from SBA, while women having higher education were 2.75 (95% CI: 1.66–4.55) times more likely to receive assistance from SBA and 2.19 (95% CI: 1.22–3.92) times more likely to attain PNC services. Moreover, compared to women with no educated husband, women having higher educated husbands were 2.58 (95% CI: 1.74–3.82) times more likely to receive SBA and 1.64 (95% CI: 1.12–2.40) times more likely to attain PNC service.

**Table 3:** Factors influencing access to and utilization of SBA and PNC services.

Variables	SBA						PNC					
	Model 1			Model 2			Model 3			Model 4		
	COR	95% CI	*P value*	AOR	95% CI	*P value*	COR	95% CI	*P value*	AOR	95% CI	*P value*
Have autonomy [*Ref*. *No*]	1.10	0.94–1.29	0.249	1.07	0.88–1.29	0.485	1.02	0.84–1.22	0.876	0.83	0.64–1.07	0.155
Negative attitude towards IPV [*Ref*. *No*]	2.11***	1.67–2.65	0.000	1.43**	1.05–1.94	0.022	1.83***	1.49–2.25	0.000	1.44***	1.12–1.84	0.004
Age [*Ref*. *age ≤ 20*]												
*21–30*				1.55***	1.14–2.11	0.005				1.03	0.73–1.45	0.872
*≥ 31*				1.86***	1.27–2.71	0.001				1.36	0.79–2.34	0.260
Education [*Ref*. *Illiterate*]												
*Primary education*				1.55**	1.03–2.33	0.036				1.01	0.67–1.54	0.950
*Secondary education*				1.81***	1.22–2.70	0.003				0.93	0.59–1.46	0.754
*Higher education*				2.75***	1.66–4.55	0.000				2.19***	1.22–3.92	0.009
Last child’s birth order [*Ref*. *one child*]												
*2–3*				0.69**	0.51–0.93	0.017				1.00	0.77–1.30	0.985
*≥ 4*				0.48***	0.36–0.65	0.000				0.85	0.62–1.17	0.313
Had pregnancy complications [*Ref*. *No*]				1.46***	1.21–1.76	0.000				1.60***	1.32–1.93	0.000
Read newspaper at least once in a week [Ref. No]				1.10	0.69–1.75	0.682				0.85	0.46–1.56	0.600
Watch TV at least once in a week [Ref. No]				1.30*	0.98–1.73	0.070				1.43***	1.12–1.83	0.004
Husband’s Education [*Ref*. *Illiterate*]												
*Primary education*				1.32**	1.02–1.70	0.036				1.33*	1.00–1.76	0.050
*Secondary education*				1.56***	1.16–2.09	0.003				1.58***	1.12–2.23	0.009
*Higher education*				2.58***	1.74–3.82	0.000				1.64**	1.12–2.40	0.011
Muslim [*Ref*. *others*]				0.73	0.49–1.10	0.135				0.73	0.46–1.16	0.188
Live in rural area [Ref. *Urban*]				0.67***	0.51–0.87	0.002				0.64**	0.45–0.93	0.019
Wealth Status [*Ref*. *Poor*]												
*Middle*				1.37*	1.00–1.90	0.054				1.05	0.75–1.47	0.784
*Rich*				2.48***	1.74–3.54	0.000				2.60***	1.77–3.83	0.000
Constant	0.30***	0.23–0.39	0.000	0.20***	0.11–0.38	0.000	1.09	0.84–1.40	0.527	1.53	0.79–2.99	0.208
Observations	4,667			3,490			4,439			3,492		

Note

*** p<0.01

** p<0.05

* p<0.1.

Exposure to mass media is a crucial influencing factor of access to and utilization of SBA and PNC. Women who watched TV at least once in a week were 1.30 (95% CI: 0.98–1.73) times more likely to receive SBA and 1.43 (95% CI: 1.12–1.83) times more likely to receive PNC services. Regarding wealth status, women from households with middle wealth were 1.37 (95% CI: 1.00–1.90) times more likely to receive SBA, while women from rich households were 2.48 (95% CI: 1.74–3.54) times more likely to receive service from SBA and 2.60 (95% CI: 1.77–3.83) times more likely to attain PNC compared to women from poor households. Women who had an experience of pregnancy related complications were more likely to attain SBA and receive PNC. Last child’s birth order of women was negatively associated with SBA, while women’s age was positively associated with SBA. This indicates women with higher birth orders of their last child were less likely to receive assistance from SBA during their most recent pregnancy and women with higher age during their most recent pregnancy were more likely to receive SBA.

### Understanding the channels of the effect of gender inequality on maternal healthcare services

To examine the effect of gender inequality on maternal healthcare services through different heterogeneous channels, we split the sample based on the category of their characteristics (Table 4). For instance, within religion, we looked at the effect of women’s autonomy and their attitude towards IPV on access to and utilization of maternal healthcare services but split them by Muslims and others. This analysis should help illuminate the channels that might be driving the differences in the effect of women’s autonomy and their attitude towards IPV between women from Muslim households and from other religions who are mostly dominated by Hindu (among the others more than 90% are Hindu followed by Buddhist and Christian). Likewise, to understand the channels of the heterogeneous effect of women’s autonomy and their attitude towards IPV, we spilt the sample depending on their place of residence (rural or urban), respondent’s education, husband’s education, watch TV, and household wealth status (Table 4). To understand the channels of the effect of gender inequality on maternal healthcare services, we performed both the simple and multivariate logistic regression models to estimate the unadjusted and adjusted effects. However, for the sake of brevity, the results of the adjusted effect are reported in the main text (Table 4) and the results of the unadjusted effect are reported in the appendix as S1 Table.

**Table 4:** Effect of gender inequality on access to five ANC services: Understanding the heterogeneous channels.

**Panel A**						
**Variables**	**Residence**		**Education of the respondent**			
	**Urban**	**Rural**	**No education**	**Primary**	**Secondary**	**Higher**
	(1)	(2)	(3)	(4)	(5)	(6)
Have autonomy [*Ref*. *No*]	0.88	1.29**	1.28	1.39	1.06*	1.04
	(0.62–1.24)	(1.04–1.60)	(0.59–2.81)	(0.94–2.07)	(0.84–1.34)	(0.61–1.77)
Negative attitude towards IPV [*Ref*. *No*]	1.09	1.50**	0.87	1.75*	1.44*	3.18**
	(0.66–1.79)	(1.00–2.24)	(0.36–2.07)	(0.96–3.20)	(0.95–2.18)	(1.24–8.15)
Constant	0.02***	0.14***	0.04**	0.11***	0.20***	1.16
	(0.01–0.07)	(0.06–0.29)	(0.00–0.49)	(0.04–0.35)	(0.09–0.45)	(0.11–12.51)
Other controls	Yes	Yes	Yes	Yes	Yes	Yes
Observations	1,260	2,232	327	837	1,828	500
**Panel B**						
**Variables**	**Religion**		**Education of her husband**			
	**Others**	**Muslims**	**No education**	**Primary**	**Secondary**	**Higher**
	(1)	(2)	(3)	(4)	(5)	(6)
Have autonomy [*Ref*. *No*]	0.76	1.23**	1.25	1.34*	0.90	1.74**
	(0.40–1.44)	(1.02–1.49)	(0.70–2.24)	(0.95–1.90)	(0.68–1.19)	(1.10–2.75)
Negative attitude towards IPV [*Ref*. *No*]	0.49	1.54**	1.09	1.18	1.77**	2.24*
	(0.19–1.24)	(1.10–2.16)	(0.51–2.33)	(0.75–1.84)	(1.05–2.99)	(0.94–5.34)
Constant	0.14**	0.07***	0.14**	0.05***	0.15***	0.09
	(0.02–0.85)	(0.04–0.13)	(0.02–0.78)	(0.02–0.17)	(0.05–0.46)	(0.00–2.08)
Other controls	Yes	Yes	Yes	Yes	Yes	Yes
Observations	284	3,208	632	969	1,239	652
**Panel C**						
**Variables**	**Watch TV**		**Household Wealth Status**			**All Sample**
	**No**	**Yes**	**Poor**	**Middle**	**Rich**	
	(1)	(2)	(3)	(4)	(5)	(6)
Have autonomy [*Ref*. *No*]	1.32*	1.05	1.58***	1.06	0.97	1.17*
	(0.98–1.78)	(0.84–1.32)	(1.13–2.22)	(0.82–1.38)	(0.64–1.47)	(0.98–1.41)
Negative attitude towards IPV [*Ref*. *No*]	1.68**	1.17	1.20	1.29	2.22*	1.42**
	(1.04–2.71)	(0.78–1.73)	(0.74–1.94)	(0.86–1.95)	(0.93–5.26)	(1.02–1.97)
Constant	0.09***	0.10***	0.12***	0.16***	0.08***	0.09***
	(0.04–0.22)	(0.03–0.37)	(0.05–0.30)	(0.05–0.51)	(0.01–0.51)	(0.04–0.19)
Other controls	Yes	Yes	Yes	Yes	Yes	Yes
Observations	1,489	2,003	1,126	1,512	854	3492

Note: Table 4 represents adjusted odds ratio (AOR) and *95% confidence intervals are in parentheses*.

*** p<0.01

** p<0.05

* p<0.1.

Our findings from both the unadjusted and adjusted analysis revealed that the effects of autonomy and negative attitude towards IPV were stronger for women living in rural areas compared to urban areas in providing access to and utilization of five required ANC services. We found that women having autonomy and lived in rural areas were more likely to receive five ANC services (AOR: 1.29, 95% CI: 1.04–1.60) compared to women who lived in rural areas without having autonomy (Column 2 in Panel A of Table 4). Similarly, our crude analysis also revealed that women having autonomy and lived in rural areas were more likely to receive five ANC services (COR: 1.20, 95% CI: 0.97–1.48) compared to women who lived in rural areas without having autonomy (Column 2 in Panel A of S1 Table). While women with negative attitudes towards IPV and lived in rural areas were more likely to receive five required ANC services (COR: 1.85, 95% CI: 1.31–2.60 and AOR: 1.50, 95% CI: 1.00–2.24) relative to women who lived in rural areas having positive attitudes towards IPV (Table 4, S1 Table). Moreover, we found that women’s negative attitudes towards IPV and lived in urban areas were more likely to receive five ANC services (COR: 2.10, 95% CI: 1.30–3.39) compared to women who lived in urban areas having positive attitudes towards IPV (Column 1 in Panel A of S1 Table). Based on the effect of rural residency, it appears that place of residence is an important driver for accessing maternal healthcare services through women empowerment. Regarding split sample by respondent’s education, women’s autonomy and their attitude towards IPV are performing a profound role in ensuring their access to five required ANC services. For instance, women with secondary education and having autonomy were more likely to attain five ANC services (AOR: 1.06, 95%: 0.84–1.34) compared to women with secondary education without having autonomy (Column 5 in Panel A of Table 4). While, for the same sample, women with negative attitudes towards IPV were 1.44 (95% CI: 0.95–2.18) times more likely to receive five ANC services compared to women with secondary education having positive attitudes towards IPV. Moreover, women with higher education and having negative attitudes towards IPV were more likely to get access to five ANC services (COR: 2.72, 95% CI: 1.08–6.85 and AOR: 3.18, 95% CI: 1.24–8.15) as opposed to women with higher education having positive attitudes towards IPV (S1 Table, Table 4). It is evident that women’s autonomy and their attitude towards IPV were performing positively for accessing maternal healthcare services through education. Similarly, husband’s education of the respondents was also playing a significant role to explain the linkage between women’s autonomy and their access to five required ANC services and the linkage between women’s negative attitude towards IPV and access to five ANC services (Panel B in Table 4).

We observed that women’s autonomy and their negative attitude towards IPV are playing a significant positive role in access to and utilization of five required ANC services for the Muslim households compared to women from other religions. We found that women from Muslim households having autonomy were more likely to attain five ANC services (COR: 1.26, 95% CI: 1.05–1.52 and AOR: 1.23, 95% CI: 1.02–1.49) relative to women from Muslim households without having autonomy, and women from Muslim households with negative attitudes towards IPV were more likely to receive five ANC services (COR: 2.08, 95% CI: 1.56–2.78 and AOR: 1.54, 95% CI: 1.10–2.16) as opposed to women from Muslim households having positive attitudes towards IPV (S1 Table, Table 4). We did not observe any significant effect of women’s autonomy and their attitude towards IPV on access to and utilization of five ANC services for the sample of other religions. The findings indicate that respondent’s religion is playing an important role to shape the effect of women’s empowerment on their access to maternal healthcare services. Watching TV (or without watching TV) was used as another important channel to play a significant role on access to and utilization of five required ANC services. Women without watching TV but having autonomy were more likely to get five ANC services (AOR: 1.32, 95% CI: 0.98–1.78) relative to women without having autonomy and not watching TV (Table 4). For the same subsample (women without watching TV), women with negative attitudes towards IPV were more likely to receive five ANC services (COR: 1.85, 95% CI: 1.22–2.82 and AOR: 1.68, 95% CI: 1.04–2.71) as opposed to women not watching TV but having positive attitudes towards IPV (S1 Table, Table 4). Moreover, women with negative attitudes towards IPV and watching TV were more likely to receive five ANC services relative to women watching TV but having positive attitudes towards IPV (Column 2 in Panel C of S1 Table). The findings from the adjusted logistic regression model revealed that gender inequality significantly influenced women’s access to maternal healthcare services for the subsample of women without access to information (not watching TV) compared to women having access to information. Besides, households’ wealth status is an important channel in providing access to maternal healthcare services through women’s autonomy and their negative attitude towards IPV. We found that, among the poor households, women’s autonomy revealed a stronger effect on access to and utilization of five ANC services. For the poor households, women having autonomy were more likely to get five ANC services (COR: 1.49, 95% CI: 1.06–1.42 and AOR: 1.58, 95% CI: 1.13–2.22) compared to women from the poor households without having autonomy (S1 Table, Table 4). While women from rich households having negative attitudes towards IPV were more likely to receive five ANC services (COR: 2.81, 95% CI: 1.13–7.02 and AOR: 2.22, 95% CI: 0.93–5.26) relative to women from rich households having positive attitudes towards IPV.

In addition to understanding the channels through which women’s autonomy and their negative attitudes towards IPV help to shape their access to and utilization of maternal healthcare services, we explored whether access to and utilization of maternal healthcare services varied by their heterogeneous characteristics. To better understand the factors that facilitate access to maternal healthcare services, we zoomed in on the place of residence, women’s education, their husband’s education, and watch TV to examine the heterogeneity (Table 5). For instance, how does respondent’s education affect their access to maternal healthcare services depending on their location (rural or urban)? In our earlier estimation, we found women living in rural areas were less likely to receive ANC services while their education was positively associated with their access to maternal health services. Therefore, to find this heterogeneous effect, we relied on the interaction between the residence of women (rural/urban) and their education level. Moreover, an interaction between education of the respondents and their husbands was estimated to find the effect of women’s education level depending on their husbands’ education. The interaction effects demonstrate some significant effect of women’s heterogeneous characteristics on access to and utilization of maternal healthcare services.

**Table 5:** Heterogeneity in factors influencing access to and utilization of five ANC services.

Variables	Five ANC Services		
	OR	P value	95% CI
**Panel A**			
Live in rural area [*Ref*. *Urban*]	0.58***	0.000	0.46–0.74
Education [Ref. *No education*]			
*Primary education*	1.32	0.208	0.86–2.04
*Secondary education*	2.50***	0.001	1.48–4.24
*Higher education*	6.07***	0.000	3.69–9.98
Watch TV at least once in a week [*Ref*. *No*]	2.01***	0.000	1.63–2.47
Constant	0.32***	0.000	0.19–0.54
Observation	3,529		
**Panel B: Interaction between place of residence and watch TV**			
Rural x Watch TV [*Ref*. *Urban x Do not watch TV*]			
*Urban x Watch TV*	3.24***	0.000	2.22–4.73
*Rural x Do not watch TV*	0.74	0.100	0.52–1.06
*Rural x Watch TV*	1.71***	0.004	1.19–2.45
Constant	0.51***	0.000	0.37–0.69
Observation	3,529		
**Panel C: Interaction between place of residence and education of women**			
Rural x Respondent education [*Ref*. *Urban x No education*]			
*Urban x Primary education*	2.32***	0.007	1.26–4.24
*Urban x Secondary education*	5.41***	0.000	3.00–9.77
*Urban x Higher education*	21.22***	0.000	10.66–42.27
*Rural x No education*	1.10	0.829	0.48–2.52
*Rural x Primary education*	1.24	0.514	0.65–2.36
*Rural x Secondary education*	2.52***	0.004	1.34–4.72
*Rural x Higher education*	5.05***	0.000	2.56–9.96
Constant	0.27***	0.000	0.15–0.49
Observation	3,529		
**Panel D: Interaction between respondent’s education and her husband’s education**			
(R) Education x (H) Education [*Ref*. *(R) No education x (H) No education*]			
*(R) No Education x (H) Primary Education*	1.25	0.627	0.50–3.14
*(R) No Education x (H) Secondary Education*	3.21**	0.021	1.19–8.64
*(R) No Education x (H) Higher Education*	2.27	0.532	0.17–29.60
*(R) Primary education x (H) No education*	1.21	0.492	0.70–2.11
*(R) Primary education x (H) Primary education*	1.67	0.229	0.72–3.85
*(R) Primary education x (H) Secondary education*	2.58**	0.017	1.18–5.62
*(R) Primary education x (H) Higher education*	1.98	0.305	0.54–7.28
*(R) Secondary education x (H) No education*	1.58	0.358	0.60–4.17
*(R) Secondary education x (H) Primary education*	2.37*	0.082	0.89–6.28
*(R) Secondary education x (H) Secondary education*	4.07***	0.000	1.87–8.89
*(R) Secondary education x (H) Higher education*	7.91***	0.000	4.17–14.99
*(R) Higher education x (H) No education*	6.03	0.175	0.45–81.30
*(R) Higher education x (H) Primary education*	5.65***	0.003	1.78–17.95
*(R) Higher education x (H) Secondary education*	5.37***	0.000	2.37–12.16
*(R) Higher education x (H) Higher education*	14.45***	0.000	6.67–31.30
Constant	0.23***	0.000	0.11–0.48
Observations	3,529		

Note

*** p<0.01

** p<0.05

* p<0.1; (R) Education means respondent’s education and (H) Education means husband’s education.

We found strong evidence from the interaction effect of education and residence on access to maternal healthcare services. The results show that heterogeneity in respondent’s education significantly affected access to and utilization of five ANC services with respect to the location where they reside. Women having higher education and living in urban areas were 21.22 (95% CI: 10.66–42.27) times more likely to receive five ANC services compared to women having no education and living in urban areas (Panel C in Table 5). In case of rural women, similar inference can be drawn. The results show that women living in rural areas were less likely to receive five ANC services. But the likelihood of receiving five ANC services significantly increased for women living in rural areas and having higher education. However, considering the effect size compared to women with higher education and living in urban areas (OR: 21.22; 95% CI: 10.66–42.27), women with higher education but living in rural areas were less likely to receive five ANC services (OR: 5.05; 95% CI: 2.56–9.96). These findings suggest that the effects of education might be stronger among women living in urban areas compared to those from rural areas. Likewise, the effect of women’s education significantly changed the likelihood of access to and utilization of maternal healthcare services depending on their husband’s education. For example, women having higher education and their husbands having primary education were 5.65 (95% CI: 1.78–17.95) times more likely to receive five ANC services whereas both the women and their spouses having higher education were 14.45 (95% CI: 6.67–31.30) times more likely to receive five ANC services compared to women and their partners with no education (Panel D in Table 5). Moreover, differences in watching TV significantly affected the access to and utilization of five ANC services with respect to their location. It has already been mentioned that women living in rural areas were less likely to receive five ANC services. But the likelihood of receiving five ANC services increased significantly for women living in rural areas and watching TV (Panel B in Table 5). These findings clearly reflect the heterogeneous effect of women on their access to and utilization of maternal healthcare services.

## Discussion

We observed that gender inequality expressed by lack of autonomy or lack of intrahousehold decision making power by women and their attitude towards IPV is associated with access to and utilization of maternal healthcare services, i.e., the number of ANC services, sufficient ANC visits, giving birth with SBA, and PNC services in Bangladesh. These findings suggest that gender inequality has a sizable effect on access to and utilization of maternal healthcare services. Women with higher autonomy were more likely to attain the required number of ANC services. Similarly, negative attitudes towards IPV (represents equality) positively influenced maternal healthcare during prenatal, delivery, and postpartum care. These two indicators of gender inequality affected the access to and utilization of maternal healthcare services in the expected direction. Though we observed the sizable effect of these two indicators of gender inequality in shaping maternal health services, the larger effect (in odds ratio) is observed for attitudes towards IPV than autonomy. In the social settings of Bangladesh, women who make at least two decisions within their households alone or jointly with their husbands regarding their healthcare, household large purchase and visit to family or relatives have a higher chance of utilizing the maternal healthcare services than those who are deprived of making such household decisions. This indicates that women’s autonomy matters to ensure their healthcare services. These results have some similarities with the findings in India [37] and Tanzania [38]. Similar to women’s autonomy, women who disagree with the questions related to women’s attitude towards IPV were more likely to ensure their maternal healthcare services. Women’s negative attitudes towards IPV signify their thought that male has no right to beat them. This attitude justifies their access to and utilization of maternal healthcare services as they are relatively more expressive and conscious about their rights. This result substantiates previous findings that women’s negative attitudes towards IPV are positively associated with receiving SBA [12, 37]. Though the government in Bangladesh has enacted the Domestic Violence Act 2010 to control violence against women, the rates of IPV are still high in the country [39]. It is reported that around two-thirds of ever-married women experienced IPV at least once during their lives [40], which is a serious public health concern for women, especially during their pregnancy [41]. A higher prevalence of IPV in Bangladesh might be the reason for the underutilization of maternal healthcare services.

The findings of our study also confirmed that women with higher education are more likely to receive ANC, assistance from SBA, and PNC services as they are more aware of maternal healthcare services. Moreover, women having an educated husband were more likely to attain required maternal health services. More importantly, the results of interaction between the education of women and their husbands revealed that the effect of the education of a woman is more profound if she had an educated husband. Therefore, better educational opportunities can act as a mediator in ensuring access to maternal healthcare services. Women with higher education may have greater autonomy at the household level and may experience more contact with health professionals about their problems without any restrictions. This study finding supports earlier literature in different study settings both in Bangladesh and abroad [34, 37, 42–47]. In a study, it is found that respondent’s education is an influential factor to receive SBA in Tanzania while this is not true for Senegal, and the possible reason for the differences could be attributed to the structural and religious settings of these two countries [38].

Women’s access to mass media has a positive effect on maternal health services. Access to information broadens their sense of receiving guidance regarding maternal healthcare services during pregnancy. The study result confirms that watching television positively affected the access to and utilization of maternal healthcare services. However, the effect of watching television depends on the difference of their residence (rural/urban). As the televisions channels in Bangladesh broadcast different programs covering the importance of maternal health services to avoid pregnancy-related complications, this might have positively contributed to provide more access to maternal health services. One of the studies in India supports this result where exposures to mass-media among women have played a significant role in accessing and utilizing health services [47]. Besides, women who lived in urban areas were more likely to receive required maternal health services. Our result confirms that urban women were more likely to attain sufficient ANC visits, assistance from SBA during delivery, and PNC after delivery. The possible reason could be higher socio-economic conditions of women in urban areas, the existence of better healthcare facilities, and greater accessibility. The result goes in line with the findings of studies in India and Mali, where the respondents of rural areas have less likelihood of attaining sufficient ANC visits, whereas the wealthy urban households have a higher probability in this regard [47, 48].

Our study also confirms that women who had experienced pregnancy complications earlier are more likely to receive sufficient ANC visits, five ANC services, SBA, and PNC services during their most recent pregnancy. A study in Ethiopia observed a similar outcome [48]. The risk aversion tendency by the women starting from prenatal to postpartum care could be attributed to this kind of precautionary behavior. Moreover, the study findings suggest that the higher birth order of the last child has a notable negative influence on access to the required ANC services and utilization of SBA during delivery. Particularly, the last child’s higher birth order reduced mother’s likelihood to receive different categories of maternal health services. Women with more than one pregnancy history were less likely to receive required and quality maternal healthcare services. Studies in different social settings found similar findings [2, 21, 47, 49–51], while a study in Zambia shows a positive association between higher birth order of the last child and sufficient ANC visits by women [23]. This heterogeneity might be the result of country-specific family planning policy and campaigns towards lowering population growth. Bangladesh is doing well in terms of family planning and lowering population growth [52], while Zambia still experiencing higher population growth because of the lower education level among women [53] and a restricted psycho-social setting [54]. From economic perspectives, women from comparatively rich households were more likely to attain required maternal healthcare services because of their higher affordability of meeting costs of those services. Previous studies have shown that households’ ability to pay for services is a significant predictor of access to maternal healthcare services [47, 55, 56]. From these findings, it can be claimed that in developing countries, majority of healthcare services are financed through out-of-pocket expenditure and the richer households are in a better position to ensure more access to quality maternal healthcare services.

As already reported, we observed a strong and statistically significant effect of gender inequality on access to and utilization of maternal healthcare services. However, the effect varies depending on heterogeneous channels in terms of women’s personal and household characteristics. Using split sample, we observed that place of residence is an important channel in shaping the effect of gender inequality on access to maternal healthcare services. One of the possible reasons behind the locational difference (rural vs. urban) is that easy access to institutional facilities in urban areas may be the cause of the weaker effect of women’s autonomy and their negative attitude towards IPV on access to maternal healthcare services. Easy access to healthcare facilities in urban areas may ensure higher access to maternal healthcare services irrespective of their gender gap at the household level. While trying to understand the effect of women’s intrahousehold bargaining power and their attitude towards IPV through the channel of religion, we observed that the effect is stronger for Muslim women in getting access to and use of maternal healthcare services. Usually, traditional religious norms which impose various restrictions on women’s mobility, intrahousehold decision making, where the constraints are often stronger for Muslim than for other religion [57]. Because of social norms for women in Muslim households and the presence of social prejudice in taking healthcare facilities from hospitals and qualified doctors restricts women to attain quality maternal healthcare services. However, those who were more aware of their intrahousehold decision making and intimate partner violence, made a big difference in accessing maternal healthcare services. Therefore, under the Muslim sample, women are experiencing inequality in ensuring access to maternal healthcare services depending on their degree of autonomy and attitude towards IPV. We found an interesting pattern to assess the effect of gender inequality on maternal healthcare services through the household’s economic perspective. For instance, women from rich households having negative attitudes towards IPV were more effective to ensure five required ANC services. While, for poor households, their attitude towards IPV was not effective, rather women’s autonomy was more effective in ensuring access to maternal healthcare services. Studies found that women from poor households were more likely to justify IPV because relatively they experienced more IPV [58, 59]. This can be explained in a way that women from poor households may seem IPV is a usual phenomenon as well as they may not be aware of their rights regarding IPV. Therefore, this might be a possible reason for the insignificant effect of IPV on access to quality ANC services in poor households. However, women from poor households enjoy more autonomy in intrahousehold decision making relative to women from middle and richer households [60]. This might be a reason for the positive role of women’s autonomy in ensuring access to maternal healthcare services.

## Strengths and limitations

We used nationally representative and comprehensive data covering multiple issues related to gender inequality and maternal healthcare which maintains strict ethical standards for protecting the privacy and confidentiality of the respondents during data collection. In addition, we employed an established analytical framework based on recent literature to address the study objective. Moreover, four separate outcome variables are used to represents access to and utilization of maternal healthcare services from four dimensions which could be a unique contribution of the study from a methodological viewpoint. Besides, we tried to cover how do women’s autonomy and their attitude towards IPV generating heterogeneous effects on access to maternal healthcare services through different channels related to individual and household characteristics. For instance, women in rural areas should be empowered more to attain greater access to maternal healthcare. However, there are few limitations and therefore requires some cautions in interpretation. For instance, the BDHS survey covered information about women’s autonomy on some limited aspects of intrahousehold decision making. Besides, the survey covered different aspects of IPV but mostly rely on their attitudes towards physical violence. Both the psychological and emotional violence that they usually experienced were not explored here. Therefore, some important aspects of gender inequality within households are not considered to explain women’s access to and utilization of maternal healthcare services.

## Concluding remarks

The evidence from our study indicates that gender inequality, expressed by the participation of women in intrahousehold decision making and their attitudes towards IPV, exists in Bangladesh. Women, deprived of expressing their opinions regarding their individual as well as household well-being and having positive attitudes towards IPV, have lower access to and utilization of required maternal healthcare services. This study has demonstrated that women have more autonomy in the household decision making process when they have the right to make decisions, whether alone or jointly with their husbands, and when they could raise their voice against the justifications of domestic violence. Finally, the results of our study revealed that women who were enjoying more equality (inequality) were more (less) likely to get antenatal care services, trained or skilled persons’ assistance during giving birth, and postnatal care services in Bangladesh. This indicates that gender inequality is a matter of ensuring higher access to and utilization of maternal healthcare services. To ensure more access to maternal healthcare services, it is needed to raise the autonomy of women and change their norms towards IPV. The country is experiencing a higher prevalence of IPV, thus, we recommend strong surveillance and monitoring by the law enforcement authority and proper implementation of the domestic violence act to reduce the prevalence of IPV which in turn could positively contribute to gender equality and improve access to and utilization of maternal healthcare in Bangladesh.

## Supporting information

S1 Table Unadjusted effect of gender inequality on access to five ANC services: Understanding the heterogeneous channels. *Note*: S1 Table represents crude odds ratio (COR) and 95% confidence intervals are in parentheses. *** p<0.01, ** p<0.05, * p<0.1.
(DOCX)
