# Supplementary material for: Does gender inequality matter for access to and utilization of maternal healthcare services in Bangladesh?
Source: PLoS One. 2021 Sep 16;16(9):e0257388. doi: 10.1371/journal.pone.0257388 (PMC8445442; doi:10.1371/journal.pone.0257388)
Supplement: S1 Table — Note: S1 Table represents crude odds ratio (COR) and 95% confidence intervals are in parentheses. *** p<0.01, ** p<0.05, * p<0.1. (DOCX) [file pone.0257388.s001.docx]

**S1 Table. Unadjusted effect of gender inequality on access to five ANC services: Understanding the heterogeneous** **channels.**

| **Panel A** | | | | | | |
| --- | --- | --- | --- | --- | --- | --- |
| **Variables** | **Residence** | | **Education of the respondent** | | | |
|  | **Urban** | **Rural** | **No education** | **Primary** | **Secondary** | **Higher** |
|  | (1) | (2) | (3) | (4) | (5) | (6) |
| Have autonomy [*Ref. No*] | 1.00 | 1.20* | 1.47 | 1.39* | 1.13 | 1.28 |
|  | 0.72 - 1.39 | 0.97 - 1.48 | 0.64 - 3.38 | 0.96 - 2.02 | 0.89 - 1.42 | 0.79 - 2.08 |
| Negative attitude towards IPV [*Ref.* *No*] | 2.10*** | 1.85*** | 1.15 | 1.97** | 1.51* | 2.72** |
|  | 1.30 - 3.39 | 1.31 - 2.60 | 0.50 - 2.63 | 1.04 - 3.74 | 0.99 - 2.31 | 1.08 - 6.85 |
| Constant | 0.69 | 0.31*** | 0.19*** | 0.19*** | 0.55*** | 0.84 |
|  | 0.39 - 1.21 | 0.21 - 0.44 | 0.09 - 0.44 | 0.10 - 0.36 | 0.36 - 0.84 | 0.34 - 2.09 |
| Other controls | No | No | No | No | No | No |
| Observations | 1,260 | 2,234 | 327 | 838 | 1,829 | 500 |
| **Panel B** | | | | | | |
| **Variables** | **Religion** | | **Education of her husband** | | | |
|  | **Others** | **Muslims** | **No education** | **Primary** | **Secondary** | **Higher** |
|  | (1) | (2) | (3) | (4) | (5) | (6) |
| Have autonomy [*Ref. No*] | 0.69 | 1.26** | 1.27 | 1.45** | 0.86 | 2.02*** |
|  | 0.35 - 1.36 | 1.05 - 1.52 | 0.68 - 2.37 | 1.02 - 2.06 | 0.65 - 1.13 | 1.32 - 3.07 |
| Negative attitude towards IPV [*Ref.* *No*] | 0.79 | 2.08*** | 1.34 | 1.31 | 2.00*** | 2.62*** |
|  | 0.32 - 1.97 | 1.56 - 2.78 | 0.66 - 2.69 | 0.87 - 1.99 | 1.23 - 3.25 | 1.31 - 5.24 |
| Constant | 1.39 | 0.33*** | 0.20*** | 0.31*** | 0.54** | 0.65 |
|  | 0.45 - 4.30 | 0.24 - 0.46 | 0.09 - 0.41 | 0.20 - 0.47 | 0.32 - 0.89 | 0.32 - 1.29 |
| Other controls | No | No | No | No | No | No |
| Observations | 284 | 3,210 | 632 | 971 | 1,829 | 500 |
| **Panel C** | | | | | | |
| **Variables** | **Watch TV** | | **Household Wealth Status** | | | **All Sample** |
|  | **No** | **Yes** | **Poor** | **Middle** | **Rich** |  |
|  | (1) | (2) | (3) | (4) | (5) | (6) |
| Have autonomy [*Ref. No*] | 1.28 | 1.13 | 1.49** | 1.07 | 1.07 | 1.20** |
|  | 0.94 - 1.74 | 0.91 - 1.42 | 1.06 - 2.08 | 0.83 - 1.39 | 0.72 - 1.61 | 1.01 - 1.43 |
| Negative attitude towards IPV [*Ref.* *No*] | 1.85*** | 1.57** | 1.29 | 1.36 | 2.81** | 1.93*** |
|  | 1.22 - 2.82 | 1.05 - 2.34 | 0.80 - 2.09 | 0.89 - 2.08 | 1.13 - 7.02 | 1.46 - 2.56 |
| Constant | 0.21*** | 0.70 | 0.17*** | 0.54*** | 0.97 | 0.37*** |
|  | 0.13 - 0.32 | 0.46 - 1.08 | 0.11 - 0.27 | 0.34 - 0.84 | 0.38 - 2.51 | 0.27 - 0.51 |
| Other controls | No | No | No | No | No | No |
| Observations | 1,491 | 2,003 | 1,128 | 1,512 | 854 | 3,494 |

Note: Table S1 represents crude odds ratio (COR) and *95% confidence intervals are in parentheses. *** p<0.01, ** p<0.05, * p<0.1*
